# Antifungal activity of linalool against fluconazole-resistant clinical strains of vulvovaginal *Candida albicans* and its predictive mechanism of action

**DOI:** 10.1590/1414-431X2022e11831

**Published:** 2022-08-15

**Authors:** C.I.S. Medeiros, M.N.A. de Sousa, G.G.A. Filho, F.O.R. Freitas, D.P.L. Uchoa, M.S.C. Nobre, A.L.D. Bezerra, L.A.D.M.M. Rolim, A.M.B. Morais, T.B.S.S. Nogueira, R.B.S.S. Nogueira, A.A.O. Filho, E.O. Lima

**Affiliations:** 1 Departamento de Ciências Farmacêuticas, Universidade Federal da Paraíba, João Pessoa, PB, Brasil; 2 Curso de Medicina, Centro Universitário UniFIP, Patos, PB, Brasil; 3 Unidade Acadêmica de Ciências Biológicas (UACB), Universidade Federal de Campina Grande, Patos, PB, Brasil

**Keywords:** Linalool, Antifungal resistance, Fluconazole, Vulvovaginal candidiasis, Mechanism of action

## Abstract

*Candida albicans* is the most frequently isolated opportunistic pathogen in the female genital tract, with 92.3% of cases in Brazil associated with vulvovaginal candidiasis (VVC). Linalool is a monoterpene compound from plants of the genera *Cinnamomum*, *Coriandrum, Lavandula*, and *Citrus* that has demonstrated a fungicidal effect on strains of *Candida* spp., but its mechanism of action is still unknown. For this purpose, broth microdilution techniques were applied, as well as molecular docking in a predictive manner for this mechanism. The main results of this study indicated that the *C. albicans* strains analyzed were resistant to fluconazole and sensitive to linalool at a dose of 256 µg/mL. Furthermore, the increase in the minimum inhibitory concentration (MIC) of linalool in the presence of sorbitol and ergosterol indicated that this molecule possibly affects the cell wall and plasma membrane integrity of *C. albicans*. Molecular docking of linalool with proteins that are key in the biosynthesis and maintenance of the cell wall and the fungal plasma membrane integrity demonstrated the possibility of linalool interacting with three important enzymes: 1,3-β-glucan synthase, lanosterol 14α-demethylase, and Δ 14-sterol reductase. *In silico* analysis showed that this monoterpene has theoretical but significant oral bioavailability, low toxic potential, and high similarity to pharmaceuticals. Therefore, the findings of this study indicated that linalool probably causes damage to the cell wall and plasma membrane of *C. albicans*, possibly by interaction with important enzymes involved in the biosynthesis of these fungal structures, in addition to presenting low *in silico* toxic potential.

## Introduction

Vulvovaginal candidiasis (VVC) is caused by abnormal yeast-like fungi growth on the female genital tract mucosa as a consequence of a series of endocrine and immunologic dysfunctions and indiscriminate and prolonged use of antibiotics (1,2). In addition, it is one of the most common conditions diagnosed in female gynecological consultations (3). Approximately 75% of this population is affected at least once in their lifetime. *C. albicans* has been reported to be the cause of symptomatic VVC in 85-95% of cases (4).

The highest incidence of CVV caused by *C. albicans* in Brazil has been reported by epidemiological studies to be 92.3% (3), in Argentina, 85.95% (5), and in Pakistan, 47.7% (6).

Antifungal azole class agents are the first drugs of choice for the treatment of VVC, which can be administered orally or topically. Polyenic antifungals, mainly nystatin, are generally used as topical treatment and azoles such as fluconazole are used orally. Currently, topical fluconazole and imidazole drugs are preferred as first-line agents; however, due to the adverse effects, high costs, and strain resistance to the antifungals, alternatives to these treatments should be considered (7). Regarding cellular mechanisms of antifungal resistance to azoles and imidazoles, several studies agree that this phenomenon is mainly due to amino acid substitutions in the pharmacological target and overexpression of the *ERG 11* gene encoding the lanosterol 14α-demethylase enzyme in *C. albicans* (8). Thus, the pharmacological study of molecules, especially natural ones with antifungal potential, may constitute a possible therapeutic alternative for the treatment of CVV (9).

Linalool is a monoterpene and the major component of the essential oil of *Lavandula angustifolia* (lavender) (24.30%), a plant of the *Lamiaceae* Martinov family native to the Mediterranean coast with known antifungal activity (10). In addition, linalool is used as a food additive and flavoring agent approved by the Food and Drug Administration (FDA), also showing antifungal activity against several species of *Candida*, *Aspergillus*, *Fusarium*, and *Penicillium*, as well as against biofilms formed by these fungi. Furthermore, this molecule has shown low *in vitro* toxicity and no genotoxicity, but is irritating to the skin and eyes (11).

Given the need for new antifungals against resistant strains of *C. albicans* and with fewer adverse effects, linalool seems to be a viable alternative. Therefore, the effects of this substance on fluconazole-resistant clinical vulvovaginal isolates were studied and insights into the elucidation of the mechanism of action of linalool were obtained through *in vitro* and molecular docking assays.

## Material and Methods

### Substances

Linalool was commercially obtained from Quinarí^®^ (Brazil) with a floral scent, molecular weight of 154.25 Da, and water solubility of 1590 mg/L at 25°C. The antifungal drugs amphotericin B (AMB), nystatin (NYS), and fluconazole (FLU) were purchased from Sigma-Aldrich^®^ (Brazil). Linalool was appropriately solubilized in 150 μL (3%) dimethyl sulfoxide (DMSO) to which 100 μL (2%) of Tween 80 was added. It was then completed with sterile distilled water (5 mL qsp) to obtain an emulsion with an initial concentration of 1024 µg/mL and serially diluted to 2 µg/mL (12,13).

### Strains

The clinical isolates of *C. albicans* used in this study belonged to the Mycotheca of the Antibacterial and Antifungal Research Laboratory of the Federal University of Paraiba, Brazil, and were: LM 37, LM 41, LM 74, LM 129, LM 157, LM 160, LM 165, LM 207, LM 230, LM 240, LM 246, and LM 319 (vulvovaginal isolates). The strains of the American Type Culture Collection (*C. albicans* ATCC^®^ 76485 and *C. albicans* SC 5314, ATCC^®^ MYA-2876™) were used as control. For use in the *in vitro* assays, fungal suspensions were prepared in 0.85% saline solution from fresh cultures and the turbidity was equivalent to 0.5 on the McFarland's standard scale, which corresponds to an inoculum of approximately 1-5×10^6^ colony-forming units per milliliter (CFU/mL) (14,15).

### Minimum inhibitory concentration (MIC)

One hundred microliters (100 µL) of liquid RPMI-1640 medium was transferred to a 96-well microdilution plate with a U-shaped bottom (Alamar, Brazil). Then, 100 µL of the linalool emulsion was dispensed in the first horizontal row of the plate and serial dilutions at a ratio of two were performed, where a 100 µL aliquot was taken from the most concentrated well to the next well, resulting in concentrations of 1024-2 µg/mL. Finally, 10 µL of the *C. albicans* inoculum suspensions was added to each well of the plate, where each column represented a fungal strain. Sterility controls with AMB, cell viability assay, and assessment of the interference of the medium used in the preparation of the linalool emulsions were also performed. The plates were incubated at 35±2°C for 24-48 h. After the appropriate incubation time, the presence (or absence) of microbial growth was visually observed (14-16). The MIC was defined as the lowest concentration of linalool that produced visible inhibition of yeast growth. The antimicrobial activity of the phytocompost was interpreted as active or non-active according to the criteria proposed by Morales et al. (17): strong/good activity (MIC: <100 µg/mL); moderate activity (MIC: >100 to 500 µg/mL); weak activity (MIC: >500 to 1000 µg/mL); and inactive/no antimicrobial effect (MIC: >1000 µg/mL).

### Minimum fungicidal concentration (MFC)

The MFC was determined after the MIC reading by taking 1 µL aliquots of the MIC, MIC × 2, and MIC × 4 from the wells where there was no visible growth (supra-inhibitory concentrations) and inoculating them into new plates containing only RPMI-1640 broth. All controls were then performed and after 24-48 h of incubation at 35±2°C, a reading was taken to assess MFC based on the controls. MFC is defined as the lowest concentration capable of causing complete inhibition of fungal growth after 24-48 h at 35°C (16,18).

### Fungal cell wall effect (sorbitol assay)

Based on the previously observed MIC and MFC results, the clinical *C. albicans* strain LM 129 and the standard *C. albicans* strain ATCC 76485 were considered representative for the subsequent assays. Therefore, the determination of the MIC of linalool in the presence of sorbitol (an osmotic protector of fungal protoplasts) was performed by microdilution in 96-well plates. To each well, 100 µL of RPMI-1640 supplemented with sorbitol of molecular weight 182.17 g (Vetec Química Fina Ltda, Brazil) was added, both at double concentration. Subsequently, 100 µL of the linalool emulsion was dispensed into the wells of the first row of the plate. Using serial dilution in the ratio of two, the required concentrations of linalool were obtained in each well with a final sorbitol concentration of 0.8 M. Finally, 10 µL of the fungal inoculum (1-5×10^6^ CFU/mL) of *C. albicans* strains (LM 129 and ATCC 76485) was added to the wells, where each column of the plate referred to a specific fungal strain (19,20). All controls were then performed as already described in the previous sections.

### Interaction with fungal cell membrane ergosterol (ergosterol assay)

The determination of the MIC of linalool against *C. albicans* strains (LM 129 and ATCC 76485) in the presence of exogenous ergosterol was performed by microdilution in 96-well plates. If the antifungal activity of linalool is caused by its binding to ergosterol, the exogenous ergosterol will prevent the monoterpene from binding to ergosterol in the fungal cell membrane. In the presence of exogenous ergosterol, linalool forms a complex with it and not with the membrane ergosterol. Consequently, there is an increase in the MIC in the presence of exogenous ergosterol compared to the control. The RPMI-1640 liquid culture medium was used with the addition of 400 µg/mL of ergosterol (Sigma-Aldrich^®^). The same procedure was carried out with AMB, whose mechanism of action is known and involves interaction with ergosterol of the fungal cell membrane to serve as a positive control of results. Growth control of the microorganism was performed with 100 µL of culture medium and ergosterol at equal concentrations and 10 µL of each standard fungal inoculum. The plates were aseptically sealed and incubated at 35±2°C for 24-48 h for later reading. Therefore, it was possible to compare the MIC values of linalool against *C. albicans* strains in the absence and presence of exogenous ergosterol (20).

### Molecular docking

The chemical structure of linalool was obtained from the NCBI PubChem ligand database (https://pubchem.ncbi.nlm.nih.gov/) and had its geometry optimized using Avogadro software (v. 1.2.0; USA), using the molecular mechanics method and the MMFF94 force field for organic molecules. The enzymes analyzed in this study were obtained from the Protein Data Bank (PDB) webpage (https://www.rcsb.org), together with their cocrystallized ligands and respective codes: 1,3-β-glucan synthase (1EQC) (1.85 Å) crystallized with castanospermine, lanosterol 14α-demethylase (ERG 11) (5TZ1) (2.00 Å) crystallized with VT-1161 (oteseconazole), and Δ 14-sterol reductase (ERG 24) (4QUV) (2.74 Å) crystallized with NADPH. The resolution of crystallographic structures deposited in PDB considered ideal to be 1.8-3.2 Å (21).

Molecular docking was performed using the free AutoDock Vina software (The Scripps Institute, USA). Protein preparation stages included removal of heteroatoms (water and ions), addition of polar hydrogens, and charge assignment. The active sites of the enzymes were delineated around the cocrystallized ligands using grid boxes of appropriate sizes. The process of docking validation was based on redocking, which consists in reflecting the position and orientation of the ligand found in the crystalline structure. Thus, the value of the root mean square deviation (RMSD) should be ≤2.0 Å. Therefore, the procedure adopted was that of molecular docking with rigid protein (with no changes in the positions of the atoms) and flexible ligands (22).

Visualization and preparation of the crystallographic structures of proteins and ligands for redocking and molecular docking were performed in PyMOL^TM^ 2.0 software (Schrödinger LLC, USA) and Discovery Studio (DS) Visualizer (v.4.1) (Accelrys Software Inc., USA).

### ADMET screening of natural compound

Linalool was submitted to online pharmacokinetics prediction tools (pkCSM - pharmacokinetics) (http://biosig.unimelb.edu.au/pkcsm/prediction) to predict its most important pharmacokinetic and toxicological properties (absorption, distribution, metabolism, excretion, and toxic effects - ADMET). These properties include absorption: Caco-2 permeability, water-solubility, human intestinal absorption, P-glycoprotein substrate, P-glycoprotein I and II inhibitors, and skin permeability; distribution: steady-state volume of distribution (Vss), unbound fraction, blood-brain barrier permeability (BHE), and central nervous system (CNS) permeability; metabolism: a substrate for P-450 isoforms; and excretion: total drug clearance and possible toxic effects (23). The free software Osiris Property Explorer (https://www.organic-chemistry.prog/peo/) was also used to indicate possible mutagenic effects, tumorigenic effects, irritability, effects on the reproductive system, and to predict the drug-related properties of linalool based on Lipinski's Rule of Five. This rule states that most ‘drug-like' molecules have cLogP ≤5, molecular weight ≤500 Da, number of hydrogen-bond acceptors ≤10 (nALH ≤10), and number of hydrogen-bond donors ≤5 (nDLH ≤5). Therefore, molecules that violate more than one of these rules may have bioavailability problems (24,25).

## Results

### Fungicidal effect of linalool against fluconazole-resistant *C. albicans* strains

The broth microdilution method was applied to determine the MIC and MFC of linalool, fluconazole, and nystatin (26). Linalool acted on fungal cells, interfering with their viability with a MIC of 64 µg/mL and a MFC between 128-256 µg/mL (Table 1). It was also found that 64.28% of the clinical strains were resistant to fluconazole and 35.71% were dose-dependently sensitive to nystatin (S-DD). Together, these results indicated that the fungal strains analyzed are sensitive to linalool and that it has a fungicidal effect. In addition, the strains used were resistant to fluconazole, and decreased sensitivity of *C. albicans* to nystatin can already be observed.

**Table 1:** Minimum inhibitory concentration (MIC) values and minimum fungicidal concentration (MFC) (µg/mL) of linalool, fluconazole, and nystatin against *C. albicans* strains by broth microdilution.

Strains	^1^Linalool				^2^Fluconazole		^3^Nystatin		GC
	MIC	MFC	MFC/MIC	Effect	MIC	MFC	MIC	MFC	
LM 37	128	256	2	Fungicidal	>1024	>1024	8	32	+
LM 41	64	128	2	Fungicidal	32	128	8	16	+
LM 74	64	256	4	Fungicidal	32	128	8	16	+
LM 129	64	128	2	Fungicidal	>1024	>1024	4	16	+
LM 157	64	128	2	Fungicidal	>1024	>1024	4	8	+
LM 160	64	256	4	Fungicidal	>1024	>1024	4	32	+
LM 165	64	256	4	Fungicidal	>1024	>1024	8	8	+
LM 207	64	128	2	Fungicidal	>1024	>1024	8	8	+
LM 230	64	128	2	Fungicidal	>1024	>1024	4	8	+
LM 240	64	256	4	Fungicidal	>1024	>1024	4	32	+
LM 246	64	256	4	Fungicidal	>1024	>1024	4	16	+
LM 319	128	128	1	Fungicidal	32	128	4	4	+
ATCC 76485	64	128	2	Fungicidal	32	64	4	16	+
SC 5314	64	256	4	Fungicidal	32	64	4	8	+

GC: growth control of the microorganism in RPMI-1640, DMSO (10%), and Tween 80 (2%), without monoterpenes or antifungals. ^1^Cutoff points: fungistatic (MFC/MIC >4) and fungicidal (MFC/MIC ≤4) (Ref. 18). ^2^Cutoff points: MIC of fluconazole ≤8 (S); 16-32 (S-DD); ≥64 (R) μg/mL, document M27-A2 (Ref. 15). ^3^Cutoff points: MIC of nystatin ≤4 (S); 8-32 (S-DD); ≥64 (R) μg/mL (Ref. 26). S: susceptible; S-DD: susceptible dose-dependent; R: resistant.

### Effect of linalool on the cell wall of *C. albicans*

Based on the previously recorded MIC and MFC results, the clinical *C. albicans* strain LM 129 and the standard *C. albicans* strain ATCC 76485 were considered representative in the analysis of subsequent results.

The *C. albicans* LM 129 and *C. albicans* ATCC 76485 strains with and without 0.8 M sorbitol (an osmotic protector of fungal protoplasts) were used to verify the possibility of linalool interacting with the fungal cell wall leading to its rupture (Table 2). The MIC of linalool for both strains increased in the presence of sorbitol indicating that this compound interferes in the viability of yeast cells through molecular mechanisms that probably involves the cell wall.

**Table 2:** Effect of linalool against *C. albicans* LM 129 and *C. albicans* ATCC 76485 in the absence and presence of 0.8 M sorbitol.

Drug	MIC (μg/mL)			
	*C. albicans* LM 129		*C. albicans* ATCC 76485	
	Absence of sorbitol	Presence of sorbitol	Absence of sorbitol	Presence of sorbitol
Linalool	64	>1024	64	>1024

### Effect of linalool on the cell membrane of *C. albicans*

Linalool was found to interfere with membrane ergosterol by mechanisms of action not yet fully elucidated (as for example, inhibition of ergosterol synthesis, direct binding of linalool to ergosterol, among other mechanisms) as its MIC increased in the presence of exogenous ergosterol (Table 3).

**Table 3:** Effect of linalool and amphotericin B against *C. albicans* LM 129 and *C. albicans* ATCC 76485 in the absence and presence of ergosterol at 400 μg/mL.

Drug	MIC (μg/mL)			
	*C. albicans* LM 129		*C. albicans* ATCC 76485	
	Absence of ergosterol	Presence of ergosterol	Absence of ergosterol	Presence of ergosterol
Linalool	64	>1024	64	>1024
Amphotericin B	0.125	>256	0.125	>256

MIC: minimum inhibitory concentration.

### Interactions of linalool with enzymes through molecular docking

**Figure 1:**
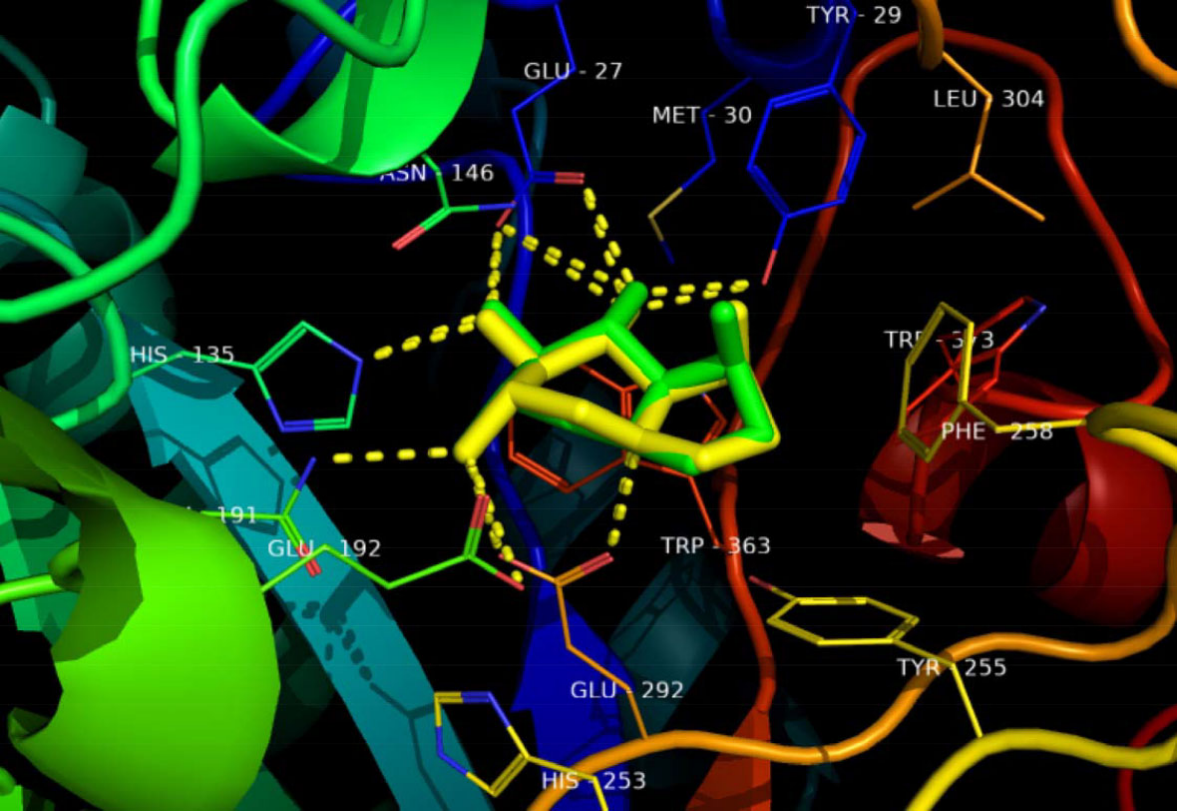
Overlapping castanospermine ligand from 1,3-β-glucan synthase with a better conformation of redocking. Green: Protein Data Bank co-crystal. Yellow: binder conformation after redocking. Dotted yellow: hydrogen-bond interactions.

**Figure 2:**
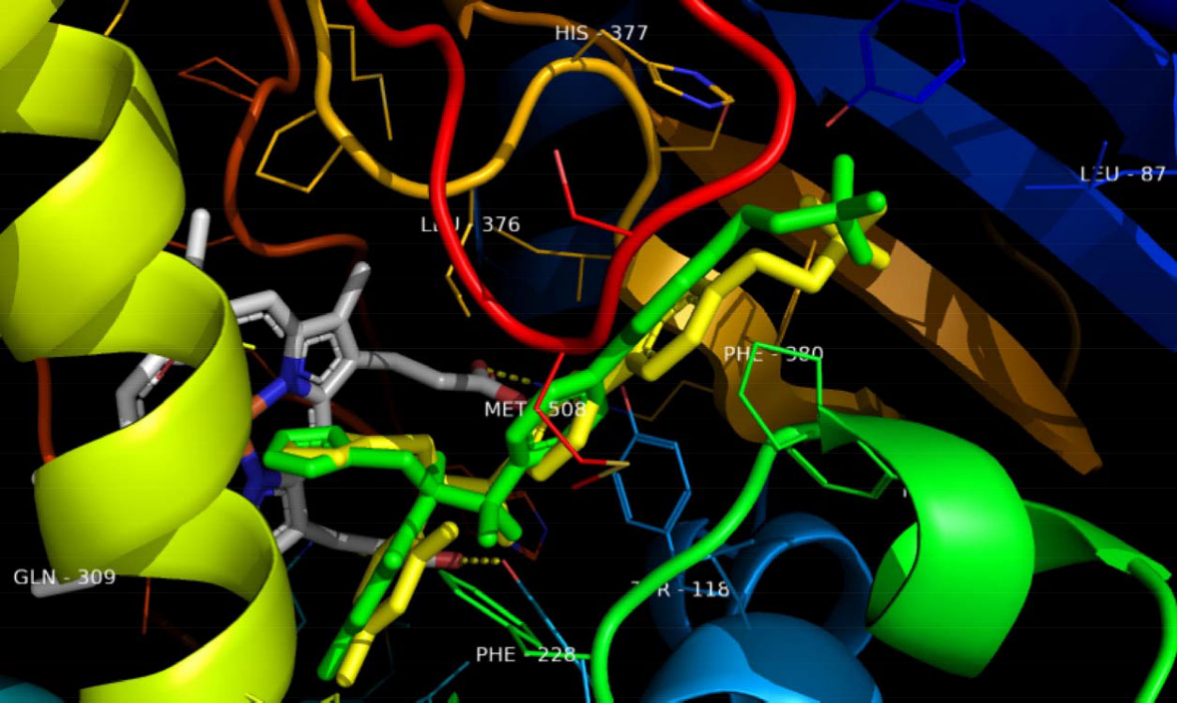
Overlapping VT-1161 (oteseconazole) ligand from lanosterol 14α-demethylase with best conformation in redocking. Green: Protein Data Bank co-crystal. Yellow: binder conformation after redocking.

**Figure 3:**
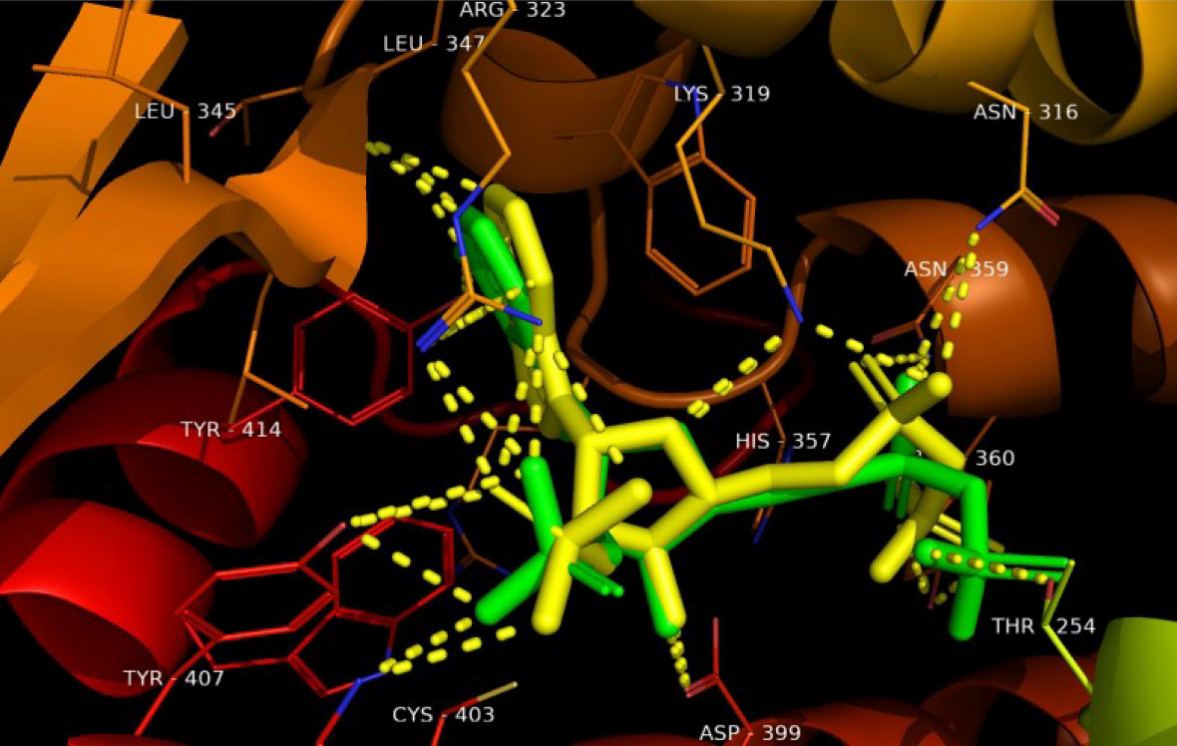
Overlapping NADPH ligand from Δ 14-sterol reductase with best conformation of redocking. Green: Protein Data Bank co-crystal. Yellow: binder conformation after redocking. Dotted yellow: hydrogen-bond interactions.

Given the possibility that linalool exerts its fungicidal effect by interfering with the cell wall and plasma membrane of fungal cells, a set of molecular docking calculations were performed with the enzymes involved in the process of biosynthesis and maintenance of these structures. Linalool was able to bind to the three enzymes analyzed with slightly different binding energies (Table 4). It can also be seen from the RMSD that the redocking was successful as these were ≤2 Å. Furthermore, Figures 1, 2, and 3 show the overlap of crystallized ligands and the redocking ligand, as well as interactions with the amino acids in the active site of each enzyme.

**Table 4:** Binding energies of Protein Data Bank (PDB) enzymes and tested compound.

Enzyme	Classification	Binding energies(kcal/mol)	RMSD (Å)	Binding energies(kcal/mol)
				Linalool
1,3-β-glucan synthesis (1EQC)	Hydrolase	-8.71	0.32	-5.70
Lanosterol 14α-demethylase (5TZ1)	Oxidoreductase	-10.93	1.30	-5.50
Δ 14-sterol reductase (4QUV)	Oxidoreductase	-13.60	0.97	-4.70

**Figure 4:**
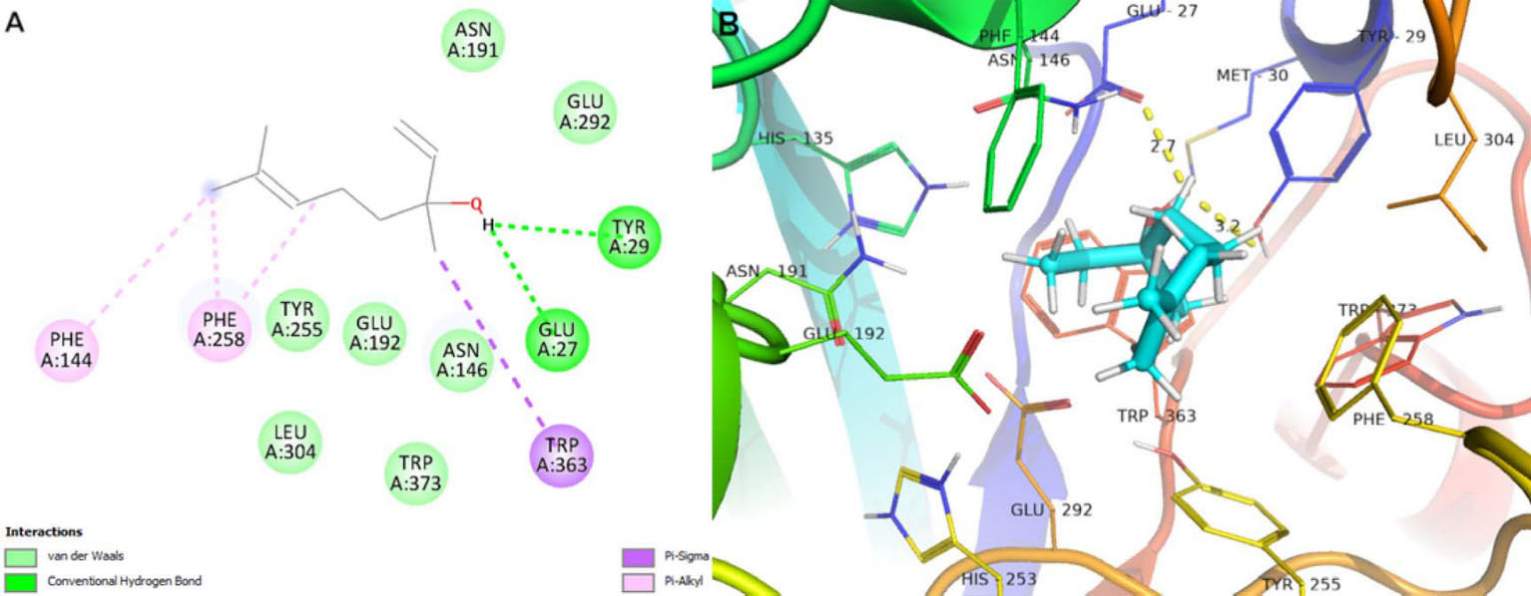
Molecular docking analysis. **A**, Two-dimensional interactions. **B**, Three-dimensional representation of linalool interactions in the 1,3-β-glucan synthase active site. Dotted yellow: hydrogen-bond interactions.

The interactions that linalool established with 1,3-β-glucan synthase via hydrogen bond, Van Der Waals, pi-sigma, and pi-alkyl interactions exhibit binding energy ΔE=-5.70 kcal/mol. The molecular complementarity of linalool with the 1,3-β-glucan synthase of the *C. albicans* cell wall was verified and, therefore, it is suggested that the inhibition of this enzyme promoted the fragility of the cell wall of these yeasts and, consequently, cell death (Figure 4).

**Figure 5:**
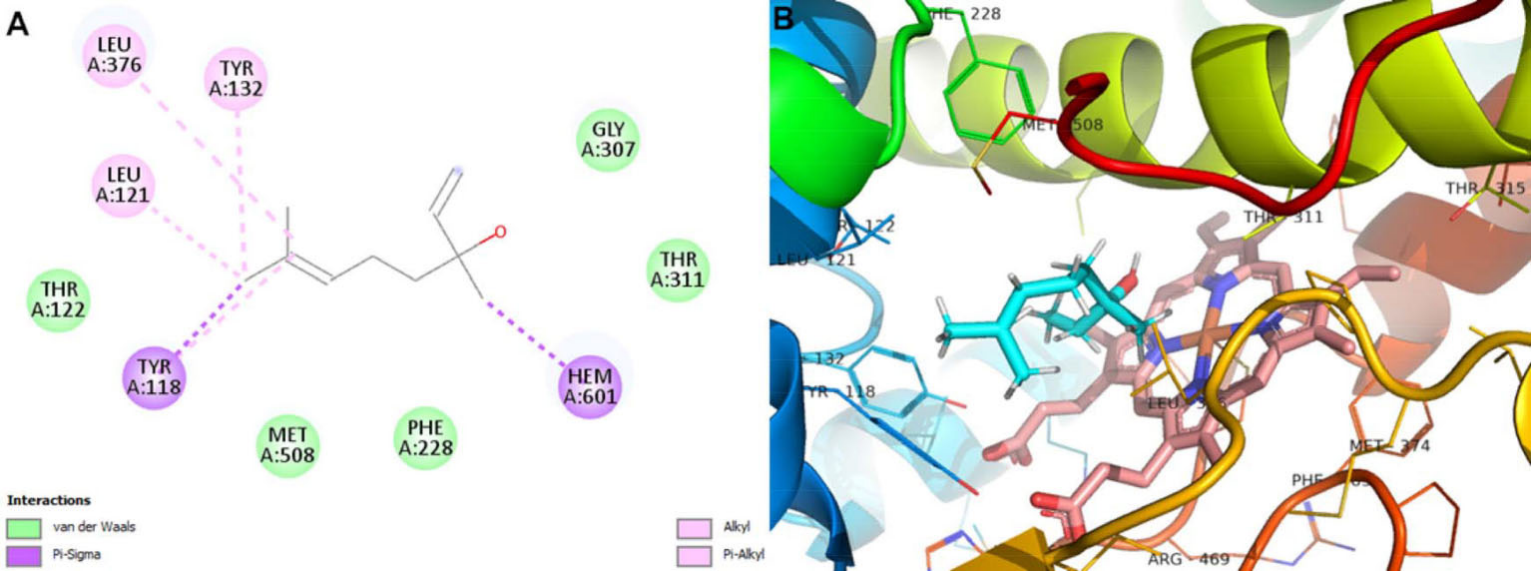
Molecular docking analysis. **A**, Two-dimensional interactions. **B**, Three-dimensional representation of linalool interactions in the lanosterol 14α-demethylase active site.

The enzyme lanosterol 14α-demethylase (ERG 11 or CYP 51) as a target of linalool showed molecular complementarity with binding energy ΔE=-5.50 kcal/mol. The CYP 51 of the fungal cell is essential for the synthesis of ergosterol; therefore, molecular interactions that cause inhibition of this enzyme may decrease the content of this sterol in the plasma membrane of *C. albicans* and cause its death (Figure 5). It was also found that the main interactions of linalool with lanosterol 14α-demethylase are Van Der Waals, pi-aigma, alkyl, and pi-alkyl interactions.

**Figure 6:**
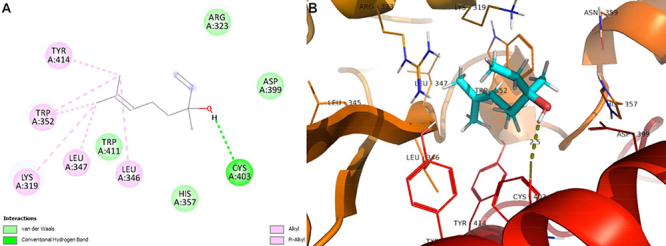
Molecular docking analysis. **A**, Two-dimensional interactions. **B**, Three-dimensional representation of linalool respective interactions in the Δ 14-sterol reductase active site. Dotted yellow: hydrogen-bond interactions.

The second stage of the conversion of lanosterol to ergosterol involves catalysis by the enzyme Δ 14-sterol reductase (ERG 24). In this study, linalool was able to bind to this enzyme with an energy of ΔE=-4.70 kcal/mol through hydrogen bond, Van Der Waals, alkyl, and pi-alkyl interactions (Figure 6).

### *In silico* ADMET study

Structure-based drug delineation is now a fairly common procedure and many potential drugs do not qualify for clinical practice due to problems found in the key pharmacokinetic parameters (ADMET). A very important class of hepatic enzymes responsible for metabolizing orally administered drugs and that have many ADMET problems are the isoforms of cytochrome P-450. Inhibition of these isoforms or the production of unwanted metabolites can result in many adverse drug reactions.

The drug analyzed showed water solubility and significant intestinal absorption, distribution, and elimination. Furthermore, inhibition of several hepatic cytochrome P-450 isoenzymes did not occur, consequently, linalool did not demonstrate hepatotoxicity in the *in silico* tests performed in this study (Table 5).

**Table 5:** *In silico* physicochemical and pharmacokinetic parameters of linalool.

Property	Model name	Predicted value	Unit
Absorption	Water solubility	-2.612	Numeric (log mol/L)
	Caco2 permeability	1.493	Numeric (log Papp in 10^-6^ cm/s)
	Intestinal absorption (human)	93.163	Numeric (% absorbed)
	Skin permeability	-1.737	Numeric (log Kp)
	P-glycoprotein substrate	No	Categorical (Yes/No)
	P-glycoprotein I inhibitor	No	Categorical (Yes/No)
	P-glycoprotein II inhibitor	No	Categorical (Yes/No)
Distribution	VDss (human)	0.152	Numeric (log L/kg)
	Fraction unbound (human)	0.484	Numeric (Fu)
	BBB permeability	0.598	Numeric (log BB)
	CNS permeability	-2.339	Numeric (log PS)
Metabolism	CYP2D6 substrate	No	Categorical (Yes/No)
	CYP3A4 substrate	No	Categorical (Yes/No)
	CYP1A2 inhibitor	No	Categorical (Yes/No)
	CYP2C19 inhibitor	No	Categorical (Yes/No)
	CYP2C9 inhibitor	No	Categorical (Yes/No)
	CYP2D6 inhibitor	No	Categorical (Yes/No)
	CYP3A4 inhibitor	No	Categorical (Yes/No)
Excretion	Total clearance	0.446	Numeric (log mL/min/kg)
Toxicity	AMES toxicity	No	Categorical (Yes/No)
	Hepatotoxicity	No	Categorical (Yes/No)

The results of the Osiris analysis showed that this monoterpene presented a low theoretical risk of toxicity (Table 6) and had considerable drug-likeness values (-6.68) and drug score (0.12). “Drug score” (combining “drug-likeness”, cLogP, cLogS, molecular mass, and toxicity risk) generates a value that infers the potential of a compound to become a future drug. Furthermore, the molecule did not have mutagenic or tumorigenic effects, nor did it have any action on the reproductive system. However, linalool has shown slight irritant potential and it is similar to pharmaceuticals, as can be seen by pharmacokinetic parameters.

**Table 6:** Toxicological properties of linalool assessed through Osiris property explorer.

Toxicological properties		Pharmacokinetic properties	
Mutagenic	N	Molecular weight (g/mol)	154.25
Tumorigenic	N	Acceptors & donors H	1.0
Irritant	Slightly toxic	Drug likeness	-6.68
Reproductive system effect	N	Drug score	0.12
-	-	Calculated lipophilicity	3.23
-	-	Calculated solubility	-2.15

N: no risk.

## Discussion

The fungal cell wall has been widely explored as a target for selective antifungal therapy. In addition, there is a significant amount of evidence that linalool exerts a fungicidal effect on *C. albicans* by interfering with its cell wall and plasma membrane (27,28), a unique structure mainly composed of chitin and glucan polymers. The cell wall and plasma membrane protect fungal cells against extracellular stress from the natural environment and the immune response of the host (29). The last class of drugs approved for clinical use were the echinocandins, which block glucan biosynthesis (30). The three echinocandin antifungal agents caspofungin, anidulafungin, and micafungin inhibit 1,3-β-glucan synthase activity, an enzyme involved in fungal cell wall synthesis. However, these drugs can be costly and require patient hospitalization due to their low bioavailability when administered orally (30). Therefore, based on the *in vitro* results and molecular docking from this study, linalool seems to exert a fungicidal effect on *C. albicans* strains by partially interacting with 1,3-β-glucan synthase.

Ergosterol is the main component of the fungal cell membrane and contributes to a variety of cellular functions, such as fluidity, membrane integrity, and the proper functioning of membrane-bound enzymes (31). Azole antifungals are the most commonly used pharmaceuticals in the clinic for the treatment of VVC and infections of other anatomical sites. They are widely used in the treatment and prevention of mycoses due to their broad-spectrum activity and because they inhibit the cytochrome P-450-dependent enzyme lanosterol 14α-demethylase (CYP51) encoded by the *ERG11* gene that converts lanosterol to ergosterol in the cell membrane, inhibiting fungal growth and replication (31). However, the use of these drugs can have some disadvantages, such as the emergence of azole-resistant strains due to selective pressure from frequent use and interaction with the cytochrome P-450 isoenzymes in the mammalian liver, which produces elevated transaminase levels and is characteristic of this class of drugs. In addition, first generation imidazoles and triazoles (clotrimazole, miconazole, cetoconazole, fluconazole, and itraconazole) are fungistatic and not fungicidal against *Candida* (32). In turn, linalool seems to be able to interfere with the ergosterol content of the plasma membrane of *C. albicans*, possibly in a similar way as polyenic antifungals such as amphotericin B and nystatin, by incorporating into membrane lipids and promoting the formation of permeable pores and cell membrane rupture, in addition to oxidative damage and fungal cell death (28,31). However, the *in vitro* results and molecular docking of this study suggested the predictive hypothesis that linalool possibly interferes with ergosterol levels by interacting with lanosterol 14α-demethylase and Δ 14-sterol reductase, in addition to affecting the cell wall of *C. albicans* by binding to 1,3-β-glucan synthase and consequently affecting cell growth (Table 4, and Figures 4 and 5).

Interestingly, the second stage in the conversion of lanosterol to ergosterol involves catalysis by the enzyme Δ 14-sterol reductase (Erg24). In contrast to Erg11, this enzyme is not a component of cytochrome P-450 in mammalian liver, suggesting that drugs against this fungal enzyme may not produce the adverse drug interactions often seen with azole drugs (31). Thus, the molecular docking of linalool with Δ 14-sterol reductase suggested a possible interaction releasing -4.70 kcal/mol of energy and possibly interfering with fungal viability (Table 4 and Figure 6).

Therefore, linalool was predictively shown to be a promising drug candidate against *C. albicans*, binding to several important targets that compromise fungal viability and exhibiting ADMET pharmacokinetic characteristics with significant theoretical oral bioavailability, low toxicity, and high similarity to pharmaceuticals (28). However, in *in vivo* studies with rabbits and rats, after rapid intestinal absorption, linalool is an enzymatic inducer of the microsomal cytochrome P-450 system, which metabolizes this monoterpene into 8-hydroxy linalool and 8-carbox linalool, which are excreted mainly via the urinary tract (33).

The therapeutic use of phytochemicals extracted from essential oils of plant origin, such as linalool, presents some limitations mainly regarding their solubility and bioavailability. In this sense, drug delivery systems may constitute versatile and alternative platforms to overcome the disadvantages of phytochemical administration, aiming at the improvement of their bioactive effects (34). Some solubilizing agents, such as dimethyl sulfoxide (DMSO), generally improve the bioavailability of linalool, but can also cause cellular toxicity and undesirable side effects. Furthermore, as a volatile compound, linalool is unstable and has a short half-life, which severely restrict its clinical application (35). Moreover, the lipophilic nature of linalool confers low solubility in water. In order to overcome these limitations, several recent studies have described the complexation of linalool with cyclodextrins (36).

Cyclodextrins are supramolecular structures characterized by the formation of a ring, and β-cyclodextrins are the most common form used in drug delivery. The β-cyclodextrins have a truncated cone shape and are composed of seven glucopyranoside units. In cyclodextrin complexes, the hydrophilic outer surface confers water solubility and the hydrophobic inner cavity allows the inclusion of lipophilic compounds such as linalool (37). Nanoscale delivery systems can also be naturally used to encapsulate linalool.

The scientific literature describes many examples of the use of lipid nanoparticles to overcome the challenges involved in the delivery and release of natural compounds, such as flavonoids, polyphenols, and carotenoids, which promote important health benefits (38). Lipid nanoparticles have a wide range of important characteristics, such as reduced particle size (between 40 and 1000 nm), large surface area, high loading capacity, possibility of controlled release of the active compound, easy large-scale production, and most importantly, a biocompatible and biodegradable nature (35,39). Thus, linalool strongly benefits from its loading into lipid nanoparticles (40), since these particles are able to overcome the physicochemical difficulties of linalool.

**Figure 7:**
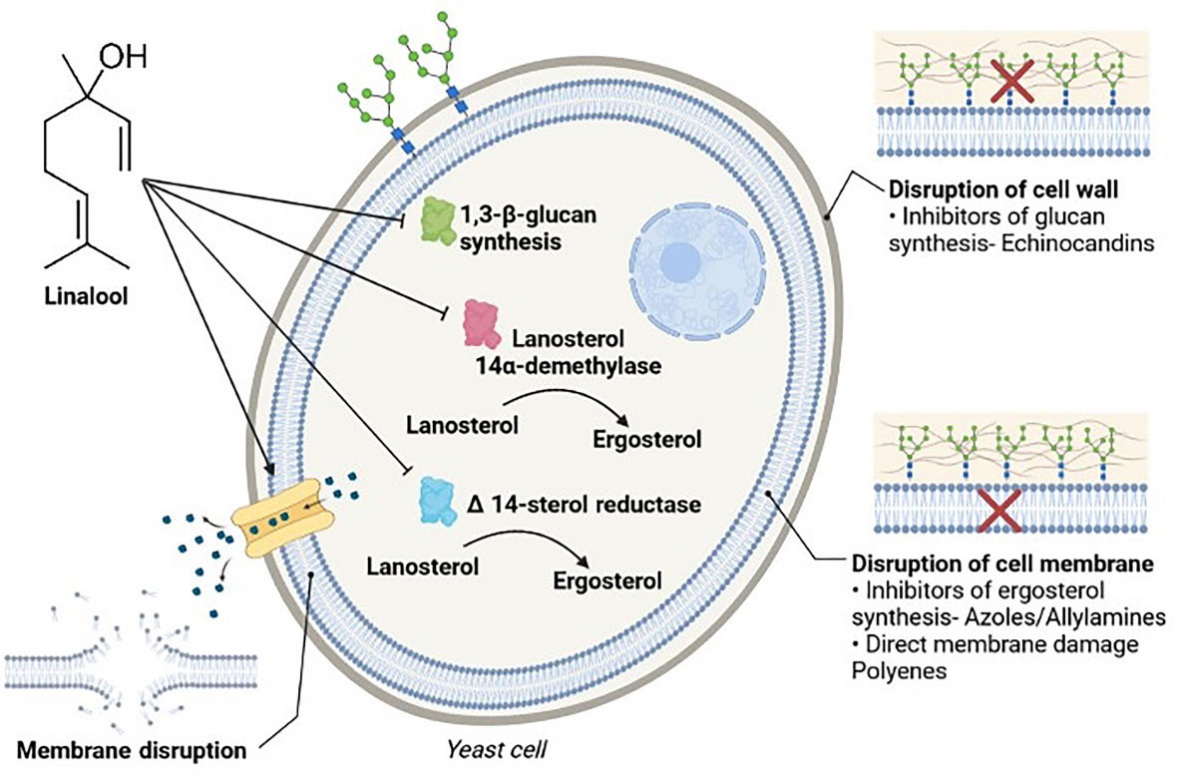
Summary representation of the predictive mechanism of action of linalool against four molecular targets of *C. albicans* strains based on *in vitro* test results and molecular docking. The linalool molecule appears to interfere with fungal cell wall maintenance involving 1,3-β-glucan synthase. Linalool can also interfere with fungal cell membrane integrity, altering the ergosterol content of these cells through interactions with lanosterol 14α-demethylase, Δ 14-sterol reductase, and/or formation of permeability pores and consequent lysis of the fungal cell membrane.

In summary, this research indicated that linalool is a fungicidal molecule against clinical strains of *C. albicans* from vulvovaginal secretions that are resistant to fluconazole. Moreover, based on the results of *in vitro* assays with sorbitol and ergosterol, linalool appeared to affect the membrane and cell wall integrity of *C. albicans*, and molecular docking suggested the predictive possibility of linalool interacting with key enzymes in the biosynthesis and maintenance pathways of these fungal structures. Furthermore, linalool showed low toxicological potential *in silico*, but *in vitro* and *in vivo* studies are needed to fully clarify the mechanism of action of this compound and provide more confidence in its use (Figure 7).
